# A grading system from health to death using routine experimental indicators based on the pre-chronic disease status theory

**DOI:** 10.1186/s12877-020-01653-1

**Published:** 2020-07-20

**Authors:** Yang Guang, Li Yuzhong, Liu Hui

**Affiliations:** College of Medical Laboratory, Dalian Medical University, Dalian, 116044 China

**Keywords:** Chronic disease, Risk assessment, Biological age, Biomarkers, Clinical chemistry

## Abstract

**Background:**

To establish a system for assessing pre-chronic disease status (PCDS) whereby changes in biomolecule levels occur before the appearance of physical damage to body organs. We based our study on the common biomarkers of aging, disease and end-of-life processes.

**Methods:**

The red blood cell count as well as levels of albumin, creatinine and aspartate aminotransferase were used as indicators for measurement. The basic premise for determining PCDS was that the measured value was outside the reference range for a healthy individual. A binary outcome was determined according to reference range given by the laboratory undertaking the measurements. The Biological Age Index (BAI) was used to ascertain PCDS.

**Results:**

The four indictors that we chose were sensitive for end-of-life and aging. The BAI score for each age group increased significantly with increasing age. The BAI score of patients with cardiac disease, cerebrovascular disease, cancer or chronic obstructive pulmonary disease were mostly higher than those in healthy age-matched people.

**Conclusion:**

A system for assessing PCDS centered on biomolecular detection and independent of the pathologic diagnosis could be effective.

## Background

**Fig. 1:**
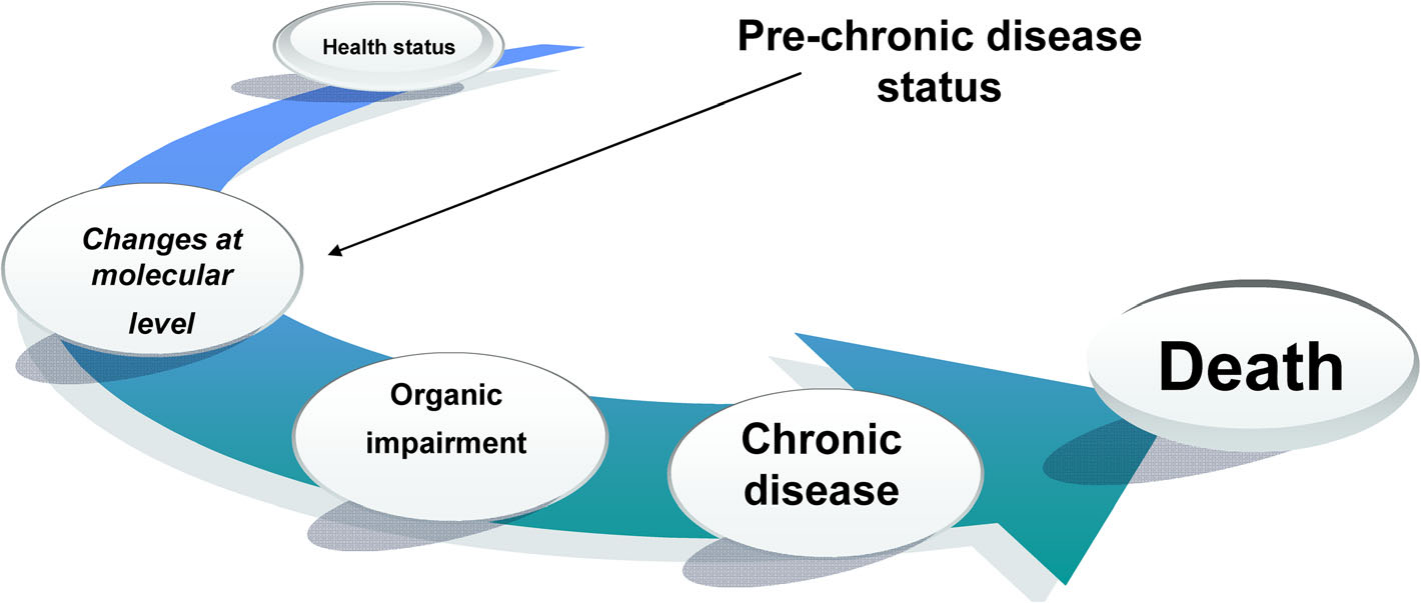
Pre-chronic disease status (schematic)

In general, it is believed that chronic diseases have no specific virulence factors and are the result of a combination of various risk factors [1–3]. The “pre-chronic disease status” (PCDS) theory refers to changes in biomolecule levels before the appearance of physical damage to body organs (Fig. 1) [4]. The PCDS could be used to aid the early diagnosis of aging-related chronic diseases.

Finding the marker molecules of PCDS is very important. As stated above, chronic diseases are the result of multiple factors, so focusing on molecules considered to be the cause of chronic diseases (e.g., lipids) may not be the best strategy. Therefore, finding marker molecules that indicate decline in organ function or mild injury to organs may be a good approach.

Chronic disease is a continuous process, which is also a key issue in its early diagnosis. Because the theory of PCDS emphasizes the status of changes in biomolecule levels before the appearance of physical damage to body organs, changes in function at the molecular level are measured [4]. There are no pathologic changes in the PCDS, but there are changes in biomolecule levels. Therefore, the understanding of chronic diseases can be advanced from monitoring of tissues and organs to measuring changes at the molecular level [5–7]. Also, unlike pathologic alterations, changes in molecular levels are largely reversible, and reflect the clinical importance of the diagnosis of PCDS. Here, we used routine experimental indicators to construct a grading system from health to death. In this way, we studied chronic diseases from a new perspective.

## Methods

### Study groups

All patients were from the Second Affiliated Hospital of Dalian Medical University (Dalian, China). All procedures involving human participants were in accordance with the 1964 Helsinki Declaration (and its later amendments) or comparable ethical standards.

#### Intensive care unit (ICU) group

The sera of patients admitted to the ICU due to chronic diseases were collected. The sera of patients who died within 2 days (35 men and 23 women; age, 72.9 ± 9.8 years) and those who died at 20–30 days (25 men and 17 women; age, 71.8 ± 10.3 years) after ICU admission were retained for experimentation. The exclusion criteria for the ICU group were accidental injuries and no record of the time of death.

#### Elderly group

Sera were collected from individuals aged > 80 years from the physical examination center (30 males and 30 females; age, 84.1 ± 2.5 years). The exclusion criteria were exacerbated episodes of various chronic diseases ≤4 weeks before the study or long-term use of medication.

#### Young group

Sera were collected from individuals  aged 20–30 years from the physical examination center (30 males and 30 females; age, 26.1 ± 2.3 years). Exclusion criteria were active infection or a history of hepatitis or other serious conditions.

#### Physiologic-variation group

Five volunteers (2 males and 3 females; age, 19–20 years) were selected and blood was collected twice at intervals of 24 h.

Eighty-three volunteers (40 males and 43 females; age, 47.3 ± 11.2 years) were selected and blood was collected twice at intervals of 30 days.

#### Verification group

We obtained data from the medical records of the physical examination center. This group of 1950 volunteers (1032 males and 918 females) was aged 20–90 years. The inclusion criteria were patients: aged > 20 years; who had visited the physical examination center for a routine health check-up rather than for specific medical treatment for ill-health; who did not have a history of taking medications on a long-term basis. The exclusion criteria were patients: with a chronic disease (e.g., coronary heart disease, cerebrovascular disease, chronic obstructive pulmonary disease (COPD), cancer); whose medical records did not have data on laboratory indicators. The active infection or a history of hepatitis or other serious conditions should also be excluded.

#### Disease group

Data were obtained from the medical records of inpatients in the Second Affiliated Hospital of Dalian Medical University. Only preoperative and pretreatment data for these patients were used.

The coronary and cerebrovascular group comprised 165 patients (92 men and 73 women, 65.7 ± 10.6 years) with coronary heart disease (as evidenced by the findings of angiography and/or computed tomography (CT) of coronary vessels), cerebral thrombosis or cerebral hemorrhage (as evidenced by the findings of magnetic resonance imaging and/or CT).

The COPD group was composed of 152 inpatients (48 men and 104 women, 68.0 ± 13.8 years). We included individuals diagnosed with COPD from stage II to IV according to Global Initiative for Chronic Obstructive Lung Disease criteria [8].

The cancer group comprised 324 cancer (gastric, liver or lung) patients (as evidenced by surgical intervention and/or pathologic findings) who underwent surgery (229 men and 95 women, 60.7 ± 10.8 years).

### Indicator selection

According to previous works [9–12], we used five criteria to choose indicators: (i) the indicator responds to aging and end of life, and its response direction (increased or decreased) is consistent with the pathologic change in direction of the indicator. (ii) the physiologic and pathologic importance of the indicator is clear; (iii) the physiologic variation of the indicator is small; (iv) the indicator is employed widely and is easy to use; (v) assay of the indicator is inexpensive.

Using the criteria stated above, we chose four indicators: red blood cell (RBC) count as well as levels of albumin (ALB), creatinine (CRE) and aspartate aminotransferase (AST).

### Blood analyses

Levels of AST (normal range: 15–40 U/L), CRE (men: 57–111 mol/L; women: 41–81 mol/L) and ALB (40–55 g/L) were measured using an automatic biochemistry analyzer (7170 s; Hitachi, Tokyo, Japan). RBCs (normal range, 4.30–5.80 × 10^12^/L) were counted using an automated hematology analyzer (SF-3000; Sysmex, Tokyo, Japan).

These investigations were carried out using standard commercial reagent kits in a clinical laboratory within Dalian Medical University. The coefficient of intra-assay variation was < 5% for each item assayed.

### Pre-disease status index (PDSI)

The PDSI was calculated based on the Frailty Index [13]. The ratio of abnormality of the indicator is defined as the PDSI. Binary outcome should be converted according to the cut-off values given by the laboratory. For example, in a study participant with an abnormality in the AST level but not of other indicators, the sum was 0 + 0 + 0 + 1 = 1. The PDSI was calculated from this sum as a percentage:

PDSI = 1/4 = 0.25.

The greater the value of the PDSI, the higher the risk of a chronic disease.

### Laboratory health index (LHI)

The mean value of the observation index (derived from the reference range of the level of an indicator given by the laboratory) was obtained. Based on the population mean of the indicator, it was divided into a healthy side and pathologic side. The ratio of the four indicators detected on the pathologic side was defined as “LHI”, and the range was 0–1. For example, in a study participant with an ALB level that was within the range but over the healthy side of mean value; other three indicators were not, the sum was 0 + 1 + 0 + 0 = 1. The LHI was calculated from this sum as a percentage:

LHI = 1/4 = 0.25.

The greater the value of the LHI, the lower the degree of health and the higher the risk of a chronic disease.

### Biological age index (BAI)

We used the BAI to represent the process from health to death. Combining the PDSI and LHI, if the PDSI > 0, the BAI was represented by PDSI + 1 and, if the PDSI = 0, the BAI was represented by the LHI. The range of the BAI was 0 to 2. Also, BAI > 1 indicated entry into PCDS.

### Statistical analyses

The independent two-sample *t*-test (measurement data) and the chi-squared test (enumeration data) were used to assess differences among groups. Analyses were undertaken using SPSS v21 (IBM, Armonk, NY, USA). *P* < 0.05 (two-tailed) was considered significant.

## Results

The raw data of the test results are shown in Table 1. Levels of the four indicators showed significant differences between 2 days and 30 days before death and between the elderly and young groups. The direction of change of all indicators was consistent with the corresponding direction of pathologic change, which suggested that the four indicators could be used for PCDS assessment.

**Table 1:** Original data for laboratory indicators in end-of-life patients and older and youth groups

Group	N	Red blood cell		Albumin		Creatinine		Aspartate aminotransferase	
		P50 (P25–P75)	P+ (%)	P50 (P25–P75)	P+ (%)	P50 (P25–P75)	P+ (%)	P50 (P25–P75)	P+ (%)
Death within 2 days	58	2.6 (2.3–3.0)	93.3	25.8 (22.9–30.8)	98.3	95.5 (54.3–235.4)	55.2	73.0 (38.3–133.0)	74.6
Death within 20–40 days	42	4.1 (3.4–4.7)	59.5	33.9 (27.5–39.6)	84.6	80.5 (56.8–114.0)	45.2	33.0 (23.8–102.0)	43.6
Age ≥ 80 years	60	4.6 (4.3–4.9)	23.3	43.5 (42.0–45.0)	6.7	72.0 (63.0–88.5)	30.0	22.5 (19.0–27.8)	6.7
Age 20–29 years	60	4.9 (4.6–5.2)	6.7	47.6 (46.4–48.6)	0	61.5 (50.5–76.0)	6.7	18.0 (16.0–24.0)	1.7
P		< 0.001	< 0.001	< 0.001	< 0.001	< 0.001	< 0.001	< 0.001	< 0.001

P+: Positive rate

PDSI data are shown in Table 2. All participants had a PDSI > 0 at 2 days before death. The probability of the PDSI being > 0 was 8.3% in the youth group. The PDSI seemed to be slightly high for a youth group. However, it was acceptable because four indicators were used in our study, so the probability of either abnormal in four indicators should be 18.5% (1–0.95^4^).

**Table 2:** Pre-chronic disease status index in different groups

Group	N	Pre-chronic disease status index				
		1.00	0.75	0.50	0.25	0
Death within 2 days	58	22 37.9%	26 44.8%	10 17.2%	0	0
Death within 20–40 days	42	5 11.9%	12 28.6%	16 38.1%	6 14.3%	3 7.1%
Age ≥ 80 years	60	0	2 3.3%	12 20.0%	10 16.7%	36 60.0%
Age 20–29 years	60	0	0	4 6.7%	1 1.7%	55 91.7%
P	–	< 0.001	< 0.001	< 0.001	< 0.001	< 0.001

BAI data for physiologic variation within 1 day and 1 month are shown in Table 3. About 50% of two test results were identical, ~ 30% of the results belonged to fluctuation level 1, and ~ 5% exceeded fluctuation level 2 (these were significant fluctuations, and suggested that health was getting better or worse).

**Table 3:** Physiologic variation for full health index within 1 day and 1 month

Result	24 h		30 days	
	N	%	N	%
Same	3	60	40	48.2
Difference of one level	2	40	26	31.4
Difference of two levels	0	0	11	13.3
Difference over two levels	0	0	6	7.0
Total	5	100	83	100

BAI scores for each age group are shown in Table 4. The BAI score for each age group increased significantly with increasing age, indicating that the BAI model was effective. BAI scores for the gender group are shown in Table 5. The BAI scores were similar between male and female groups, indicating that the BAI model was suitable for men and women. The observed population was not derived from building of the model, so the validity of the BAI could be verified.

**Table 4:** Biological age index and rate in different levels of health and pre-chronic disease in age groups

Age	N	Mean ± SD	Pre-chronic disease (%)				Health (%)					P
			2.00	1.75	1.50	1.25	1	0.75	0.50	0.25	0	
20-	170	0.51 ± 0.31	0	0	2.4	5.3	0.6	18.2	41.2	25.9	6.5	
30-	328	0.61 ± 0.31	0	0	1.2	9.1	5.5	23.2	41.5	17.1	2.4	
40-	386	0.64 ± 0.30	0	0.3	1.6	8.5	3.9	30.1	40.9	13.5	1.3	
50-	295	0.70 ± 0.35	0	0	2.4	15.9	7.5	24.1	35.3	13.6	1.4	< 0.001
60-	117	0.70 ± 0.33	0	0	1.7	15.4	5.1	29.1	35.9	12.0	0.9	
70-	436	0.85 ± 0.37	0	0.7	5.5	26.6	6.9	27.1	26.4	6.7	0.2	
≥80	218	0.95 ± 0.38	0.5	1.8	12.8	23.4	12.4	27.1	17.9	4.1	0

**Table 5:** Biological age index in gender groups

Group	N	Mean	SD	*t*	P
Men	1032	0.727	0.397	1.194	0.233
Women	918	0.707	0.313		

**Fig. 2:**
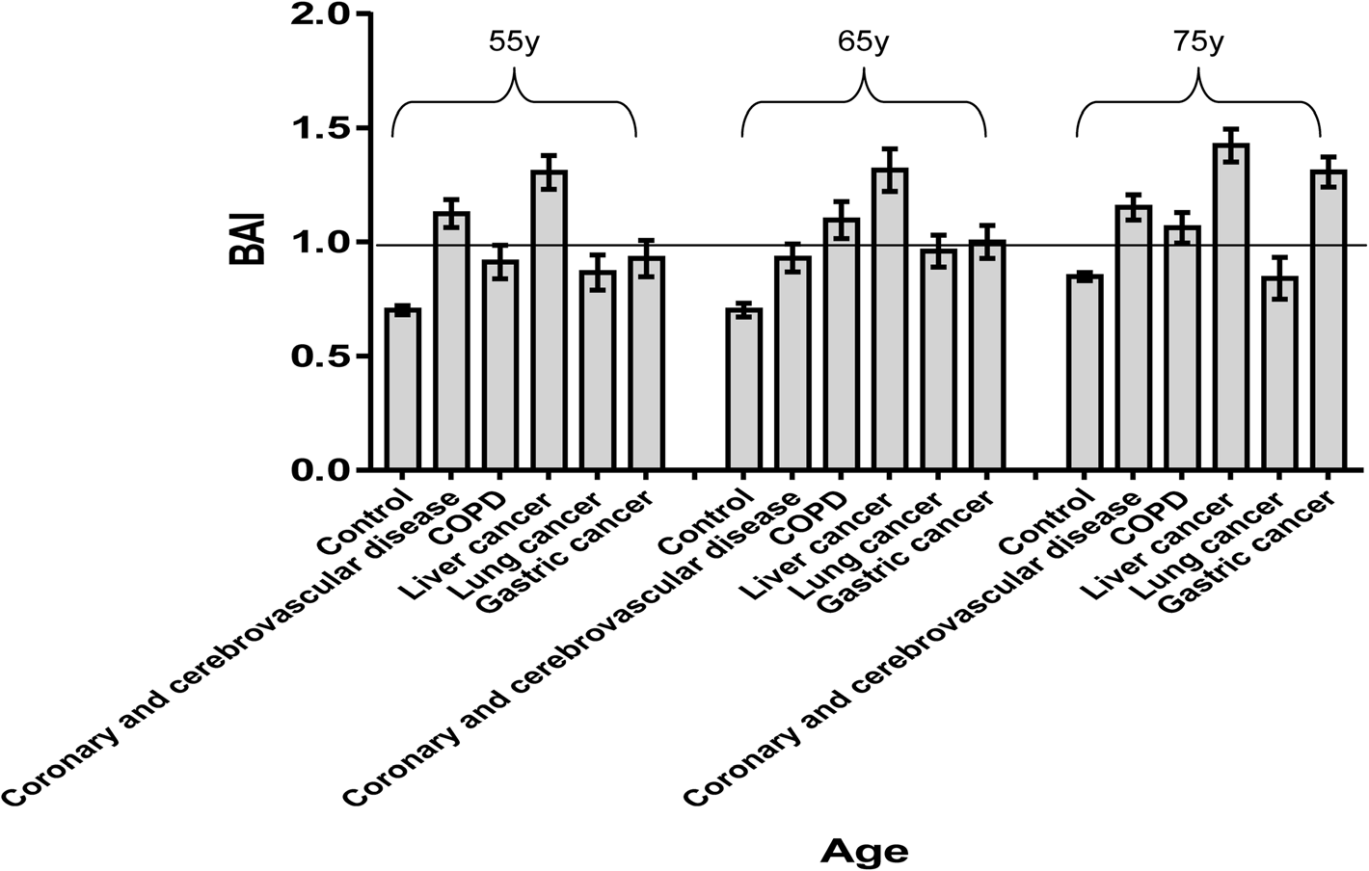
The Biological Age Index (BAI) for various diseases in same age group BAI scores in various diseases were mostly higher than those in the healthy group for the same age (although some diseases had a BAI < 1).

BAI data for various diseases are shown in Table 6. The mean BAI score for each disease group was close to or > 1.0. BAI scores in various diseases were mostly higher than those in the healthy group for the same age (Fig. 2). These data suggested, to a certain extent, that the BAI could be used to assess the risk of chronic diseases in healthy people.

**Table 6:** Biological age index and rate at different levels of health and pre-chronic disease in major chronic disease groups

Disease	Age	N	Mean ± SD	Pre-chronic disease (%)					Health (%)			
				2.00	1.75	1.50	1.25	1.0	0.75	0.50	0.25	0
Coronary and cerebrovascular disease	< 60	46	1.13 ± 0.41	0	6.5	19.6	43.5	2.2	10.9	10.9	6.5	0
	60-	56	0.93 ± 0.45	0	7.1	5.4	37.5	0	8.9	33.9	7.1	0
	70-	46	1.15 ± 0.37	0	4.3	28.3	34.8	2.2	17.4	13.0	0	0
	≥80	17	1.38 ± 0.40	0	17.6	58.8	11.8	0	0	5.9	5.9	0
Chronic obstructive pulmonary disease	< 50	15	1.02 ± 0.48	0	0	20.0	46.7	0	6.7	6.7	20.0	0
	50-	34	0.91 ± 0.43	0	0	14.7	32.4	2.9	11.8	29.4	8.8	0
	60-	26	1.10 ± 0.41	0	0	30.8	34.6	3.8	7.7	19.2	3.8	0
	70-	40	1.06 ± 0.42	0	2.5	25.0	32.5	2.5	17.5	12.5	7.5	0
	≥80	37	1.34 ± 0.36	0	18.9	35.1	32.4	0	8.1	2.7	2.7	0
Liver cancer	< 50	21	1.07 ± 0.62	0	23.8	19.0	19.0	0	4.8	9.5	19.0	4.8
	50-	32	1.30 ± 0.42	0	15.6	40.6	28.1	0	3.1	9.4	0	3.1
	60-	27	1.31 ± 0.48	0	33.3	25.9	18.5	0	3.7	14.8	3.7	0
	≥70	13	1.42 ± 0.25	0	15.4	53.8	23.1	0	7.7	0	0	0
Lung cancer	< 60	43	0.87 ± 0.50	0	4.7	7	37.2	2.3	4.7	23.3	16.3	0
	60-	50	0.96 ± 0.50	0	6.0	14.0	36.0	0	6	26.0	8.0	0
	≥70	25	0.84 ± 0.46	0	0	20.0	20.0	0	4.0	48.0	8.0	0
Gastric cancer	< 60	41	0.93 ± 0.51	0	2.4	22.0	29.3	0	2.4	26.8	14.6	2.4
	60-	41	1.00 ± 0.46	0	0	29.3	26.8	0	12.2	22.0	9.8	0
	≥70	31	1.31 ± 0.37	0	9.7	48.4	25.8	0	3.2	12.9	0	0

## Discussion

The PCDS theory conceptualizes an aging-related chronic disease as a continuous state. Our data for major lethal diseases (cardiovascular and cerebrovascular diseases, COPD and cancer) suggest that the initial stage and end stage of this continuous process were similar because of changes in levels of the four indicators (RBC count as well as levels of ALB, CRE and AST). We believe that the specific occurrence of a disease in the midst of this continuous process is the result of the cumulative effect of external factors and genetic factors, so the occurrence of specific diseases is random, and random damage will be manifested as multiple-organ damage.

The four indicators we selected were related to liver function, kidney function, hematopoiesis function and integrity of tissue, and the physiologic variations were small. Moreover, the four indicators were, theoretically, independent of each other. Once there is a simultaneous pathologic fluctuation, the disease risk is increased. Therefore, although the measured value did not lie outside the reference range, the fluctuation of multiple indicators simultaneously pointed to the direction of disease and could be considered to be the risk of disease occurrence. This view was validated in different age groups. For example, in the 70-year-old group, most of the indicators were concentrated on the pathologic side of the mean value whereas, in the 30-year-old group, most of the indicators were concentrated on the healthy side of the mean value.

Another concern was that some medications, such as antibiotics, may affect liver function [14–16] and kidney function [17, 18]. Therefore, patients who take antibiotics and use medications long-term should be excluded. PCDS does not mean that a disease is already present, so a medication is not often used in PCDS. In practical terms, the influence of antibiotic use should be limited in our system.

Although the BAI does not denote a specific chronic disease, it can represent a biological age for an individual; aging brings with it increased risks for disease. We showed that the association between aging and disease was > 80% [19], so the experimental indicators related to aging (whether they be the cause or the result of the disease) could be used as an indicator for aging-related disease status. Results showed that the BAI score was higher in the disease group than that in the control group. However, the positive rate (instances when the BAI > 1) was not 100%. The explanation for this phenomenon is that diseases with typical changes have stopped. Hence, patients with the early stage of the disease or relatively healthy patients are observed in the surviving population, and it is necessary to observe them in the population before they die. At the end of the disease, the positive rate of the BAI was 100%, which confirmed our interpretation to some extent. For the same reason, it is also necessary to further grade “health status” with BAI score.

PCDS theory states that changes in organ function from micro-injury may be compensated. We found that if a measured value was within the reference range, there remained a large influence in physiologic variation, and that this variation might also be the result of damage. However, if the measured value was within the reference range, we still considered it as a health status or an ill-health status rather than a PCDS. The basic factor for determining PCDS should be that the measured value was outside the reference range [20]. Hence, if someone was determined to have PCDS with a BAI ≥1–2, the BAI could be targeted for interventions that may slow or prevent the chronic disease, or could be an indicator for evaluating clinical actions and intervention methods. This strategy could make medical professionals more aware of the repercussions of their medical decisions at the molecular level at the early stage of disease rather than at the stage of histopathologic impairments. An additional practical feature of our system was that it could provide a biomarker to evaluate the effectiveness of health administration objectively and promptly.

We emphasize employment of routine experimental indicators in our system for their generalizability. In all laboratories, close attention must be paid to the upper and lower limits of the reference range as well as the mean levels of the selected indicators, and adjustment for sex and ethnic groups must be made (if necessary). Therefore, our system could be generalized to other populations by adjusting the limits of the reference. Further investigation on the limits of the reference in different ethnic groups is needed to establish the appropriate system for a certain population.

## Conclusions

PCDS theory emphasizes that changes at the molecular level are not limited to the pathologic changes observed in disease. Even if the pathologic changes have reached the criteria for the diagnosis of a disease, if the compensatory function of an organ is strong, then body function can maintain a normal state, and there is no serious problem in PCDS theory. From this viewpoint, it is more meaningful to examine the function of the organ than to examine changes in the morphology of the organ. A system for assessing PCDS centered on biomolecular detection and independent of the pathologic diagnosis could be effective.

It is well known that test indicators with levels outside the reference range are considered “abnormal”. Therefore, each laboratory should pay close attention to the upper or lower limits of the reference range and mean levels of indicators. This will become the basis of using PCDS theory into practice. In this way, it may be possible to develop a new field of laboratory-based medicine.

## Data Availability

The datasets used and analysed during the current study are available from the corresponding author on reasonable request.

## Abbreviations

ALB: Albumin
AST: Aspartate aminotransferase
BAI: Biological Age Index
CRE: Creatinine
COPD: Chronic obstructive pulmonary disease
ICU: Intensive care unit
LHI: Laboratory Health Index
PCDS: Pre-chronic disease status
RBC: Red blood cell
